# Evaluating environmental DNA detection of a rare fish in turbid water using field and experimental approaches

**DOI:** 10.7717/peerj.16453

**Published:** 2024-01-02

**Authors:** Ann E. Holmes, Melinda R. Baerwald, Jeff Rodzen, Brian M. Schreier, Brian Mahardja, Amanda J. Finger

**Affiliations:** 1 Genomic Variation Laboratory, University of California, Davis, Davis, California, United States; 2 Graduate Group in Ecology, University of California, Davis, Davis, California, United States; 3 California Department of Water Resources, West Sacramento, California, United States; 4 Genetics Research Laboratory, California Department of Fish and Wildlife, Sacramento, California, United States; 5 Bureau of Reclamation, US Department of the Interior, Sacramento, California, United States

**Keywords:** Conservation, Delta smelt, Endangered species, Estuary, Environmental DNA, Particulate matter, Real-time polymerase chain reaction, Turbidity

## Abstract

Detection sensitivity of aquatic species using environmental DNA (eDNA) generally decreases in turbid water but is poorly characterized. In this study, eDNA detection targeted delta smelt (*Hypomesus transpacificus*), a critically endangered estuarine fish associated with turbid water. eDNA sampling in the field was first paired with a trawl survey. Species-specific detection using a Taqman qPCR assay showed concordance between the methods, but a weak eDNA signal. Informed by the results of field sampling, an experiment was designed to assess how turbidity and filtration methods influence detection of a rare target. Water from non-turbid (5 NTU) and turbid (50 NTU) estuarine sites was spiked with small volumes (0.5 and 1 mL) of water from a delta smelt tank to generate low eDNA concentrations. Samples were filtered using four filter types: cartridge filters (pore size 0.45 μm) and 47 mm filters (glass fiber, pore size 1.6 μm and polycarbonate, pore sizes 5 and 10 μm). Prefiltration was also tested as an addition to the filtration protocol for turbid water samples. eDNA copy numbers were analyzed using a censored data method for qPCR data. The assay limits and lack of PCR inhibition indicated an optimized assay. Glass fiber filters yielded the highest detection rates and eDNA copies in non-turbid and turbid water. Prefiltration improved detection in turbid water only when used with cartridge and polycarbonate filters. Statistical analysis identified turbidity as a significant effect on detection probability and eDNA copies detected; filter type and an interaction between filter type and prefilter were significant effects on eDNA copies detected, suggesting that particulate-filter interactions can affect detection sensitivity. Pilot experiments and transparent criteria for positive detection could improve eDNA surveys of rare species in turbid environments.

## Introduction

Environmental DNA (eDNA) is an efficient tool for surveying rare species and can indicate the presence of rare targets not detected by other methods (*e.g*., Budd et al., 2021; Renan et al., 2017). Rare targets include species of conservation concern such as endangered and invasive species. eDNA sampling usually poses little or no risk of sampling-related mortality or stress to both target and non-target organisms, an advantage when sampling endangered species or sensitive habitats. eDNA sampling may be preferred in potentially hazardous conditions (*e.g*., high gradient streams or windy weather) because water may be collected more quickly and with less risk to personnel compared with direct sampling of organisms. Despite the advantages of eDNA detection, environmental conditions may decrease detection success, especially for rare targets (*e.g*., Day et al., 2019; Egeter et al., 2018; Williams, Huyvaert & Piaggio, 2017).

eDNA detection sensitivity is influenced by interacting suites of biological and environmental conditions, described as the “ecology” of eDNA (Barnes & Turner, 2016). Target organism biomass, individual body size, and eDNA shed rate may influence eDNA detection probability (*e.g*., Sassoubre et al., 2016). Environmental conditions such as turbidity, temperature, pH, salinity, solar radiation, water movement, or microbial community composition (or the related biotic conditions) also affect species detection (*e.g*., Collins et al., 2018; Jane et al., 2015; Laramie et al., 2015; Seymour et al., 2018; Shogren et al., 2018; Strickler, Fremier & Goldberg, 2015; Tsuji et al., 2019). Turbidity is a previously recognized challenge for eDNA sampling and appears to reduce detection sensitivity and increase false negative detections (Egeter et al., 2018; Williams, Huyvaert & Piaggio, 2017).

Turbidity is a measure of light scatter and is associated with reduced water clarity. High turbidity results from a variety of unrelated phenomena including particulates spanning a range of sizes and compositions (*e.g*., sediment, inorganic material, or organic material such as plankton and plant detritus) and decreased water clarity without particulates (*e.g*., staining by tannins from plant material). Turbidity can negatively affect eDNA detection by clogging filters or introducing PCR inhibitors (*e.g*., humic compounds; Matheson et al., 2010). PCR inhibition may be mitigated using appropriate extraction methods (Hunter et al., 2015) or a post-extraction inhibitor removal step (Williams, Huyvaert & Piaggio, 2017).

Particulate matter may clog filters, leading to reduced sampling volumes, long filtration times, and decreased detection sensitivity (Day et al., 2019; Egeter et al., 2018; Li et al., 2018; Stoeckle et al., 2017; Williams, Huyvaert & Piaggio, 2017). Species detection sensitivity can be positively correlated with volume of water sampled (Schultz & Lance, 2015; Hunter et al., 2019; Sepulveda et al., 2019; Schabacker et al., 2020; Song et al., 2020). Long filtration times may negatively affect species detection by increasing pressure on the filter membrane and decreasing retention of eDNA (Thomas et al., 2018). Filters with larger pores are recommended to increase water volume and decrease filtering time in turbid systems (Turner et al., 2014; Robson et al., 2016). While some results suggest that adding an additional filtration step before the capture membrane using a filter with a larger pore size (prefiltration) increases eDNA capture (Takahara et al., 2012), others are inconclusive (Majaneva et al., 2018). A better understanding of eDNA detection dynamics in turbid water is necessary for optimizing protocols and interpreting data.

### Study species and habitat

**Figure 1:**
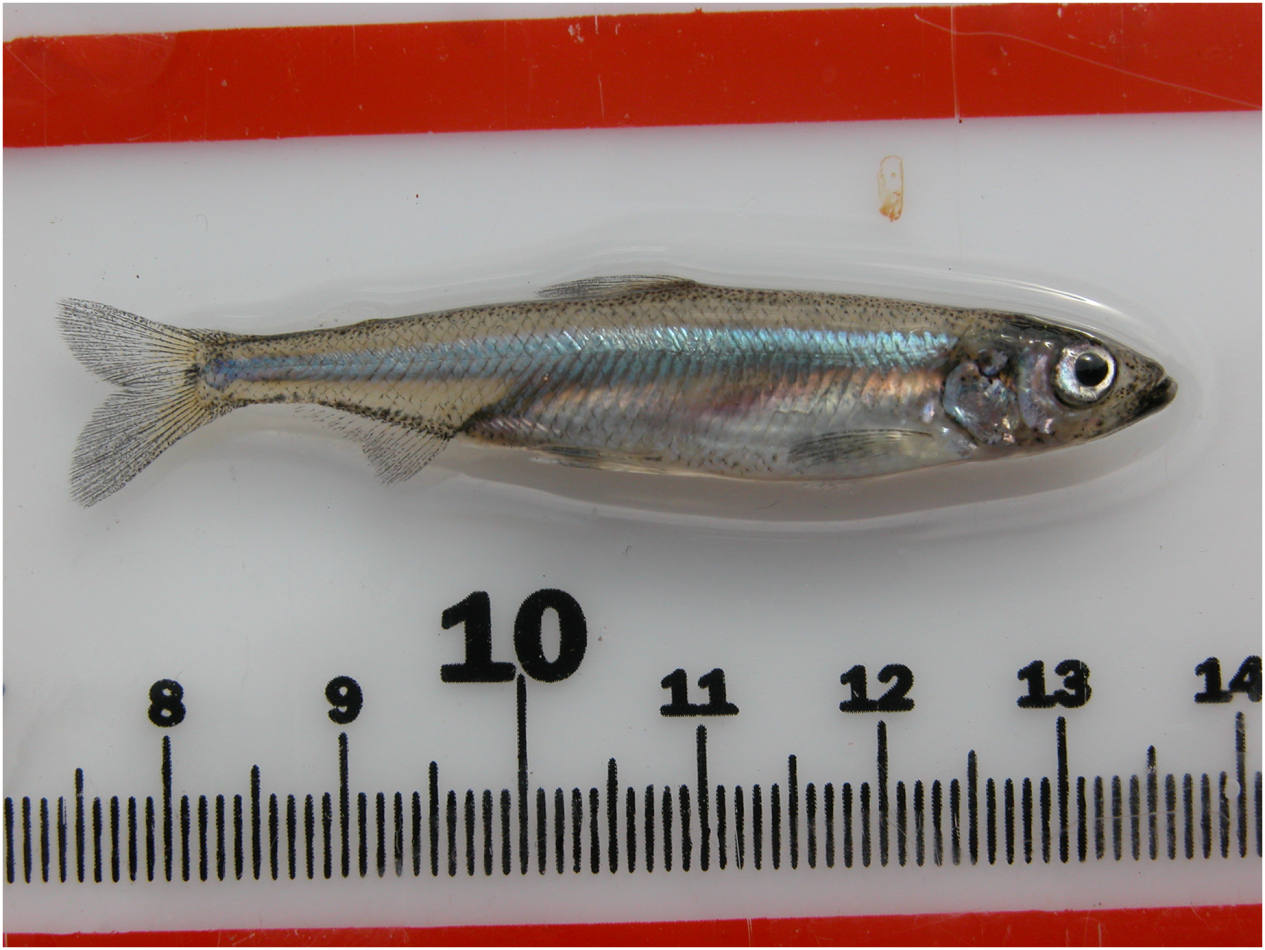
Delta smelt (*Hypomesus transpacificus*). Adult delta smelt measure approximately 5 to 7 cm. © 2007 by U.S. Fish and Wildlife Service (License: CC BY 2.0, https://www.flickr.com/photos/usfws_pacificsw/5159686356)

This study examines the effects of turbidity and filtration methods on the sensitive detection of delta smelt (*Hypomesus transpacificus;* Fig. 1), a small, critically endangered fish endemic to the San Francisco Estuary (SFE), CA, USA. Historically one of the most common species in the SFE (Erkkila et al., 1950), delta smelt are significant in indigenous MiwkoɁ (Miwok) traditional cultural practice and law (Hankins, 2018). Delta smelt were first described as semianadromous annual fish capable of spawning only once (Moyle et al., 1992; Sommer et al., 2011), but non-migratory (Hobbs et al., 2019; Campbell et al., 2022) and iteroparous (Kurobe et al., 2016) phenotypes have also been documented.

Delta smelt is considered a sentinel species of the SFE ecosystem and at imminent risk of extinction (Moyle, Hobbs & Durand, 2018). Dramatic population declines were documented prior to and following Federal Endangered Species Act (ESA) and California Endangered Species Act (CESA) protections (U. S. Fish and Wildlife Service, 1993; Sommer et al., 2007) and contemporary numbers represent less than 1% of the historical population (Moyle et al., 2016; Hobbs et al., 2017). The species appears to be especially sensitive to changes in environmental conditions including water quality, habitat degradation, and the effects of introduced species (Moyle et al., 1992; Sommer et al., 2007; Moyle et al., 2016).

Turbidity is a key feature of the environment during a critical period of delta smelt life history. Turbidity increases are significantly correlated with total river inflow, usually during winter storms, that initiate migration from brackish regions to freshwater spawning grounds in the upper estuary (Grimaldo et al., 2009; Sommer et al., 2011). Delta smelt presence is positively associated with turbid water (Feyrer, Nobriga & Sommer, 2007; Grimaldo et al., 2009; Sommer et al., 2011) perhaps due to decreased predation risk (Ferrari et al., 2014; Bennett & Burau, 2015) and increased larval feeding rates (Baskerville-Bridges, Lindberg & Doroshov, 2004; Tigan et al., 2020). Physiological performance of delta smelt is negatively affected by turbidity below 25 NTU and above 80 NTU (Hasenbein et al., 2016). Turbidity in delta smelt habitat is attributed to suspended sediment transported from upstream sources or resuspended in the water column due to wind or turbulence (Schoellhamer, 2002) and may have negative consequences for eDNA detection.

Delta smelt are monitored primarily through the Enhanced Delta Smelt Monitoring program (EDSM), an exhaustive year-round trawl and net survey conducted by the U.S. Fish and Wildlife Service (Mahardja et al., 2021; U. S. Fish and Wildlife Service et al., 2019; File S1). Given low trawl detection rates of delta smelt, sensitive and non-invasive survey methods are urgently needed.

### Objectives

This study examines the effects of turbidity and filtration methods on eDNA detection of a very rare species. The aims of this study were to assess (1) eDNA detection success of a turbidity-associated species in its natural habitat and (2) the influence of high turbidity conditions and filtration methods on eDNA detection success in an experimental setting. Experiments used high and low turbidity water collected from the SFE and tested four filter types and the addition of a prefiltration step (Table 1).

**Table 1:** Description of filters used in the filtration experiment.

Filter	Abbreviation	Filter type	Pore type	Pore size (µm)
Glass fiber (47 mm; Whatman, Cytiva, Little Chalfont, UK)	GF	Depth	Nominal	1.6
Polycarbonate track-etched (47 mm; MilliporeSigma, Burlington, MA, USA)	PC	Screen	Absolute	10
Polycarbonate track-etched (47 mm; MilliporeSigma, Burlington, MA, USA)	PC	Screen	Absolute	5
Sterivex polyvinylidene fluoride (cartridge; MilliporeSigma, Burlington, MA, USA)	ST	Screen	Absolute	0.45
**Prefilter**				
Nylon net (47 mm)	NN	Screen	Mesh	20

## Materials and Methods

Portions of this text were previously published as part of a preprint (https://doi.org/10.1101/2022.08.24.502857). Prior to all eDNA sampling, collection bottles and supplies were sterilized for at least 20 min in 20% bleach solution then rinsed three times with deionized water. Filtration used a Geopump Peristaltic Pump (Geotech, Denver, CO, USA) and silicon tubing. Tubing exterior was sterilized as above, then 20% bleach solution was pumped through the tubing for at least 1 min then flushed with deionized water for at least 1 min. Genetics work was conducted in a dedicated eDNA laboratory space following recommended guidelines including rigorous decontamination procedures, dedicated hoods used only for DNA extraction or quantitative PCR (qPCR) reaction preparation, and a separate laboratory room for PCR amplification (Goldberg et al., 2016).

### Assay performance

The presence of delta smelt DNA was assessed using a species-specific qPCR assay (Table 2; Baerwald et al., 2011). The assay was previously tested for cross-reactivity with the two cooccurring smelts (Actinopterygii: Osmeridae) and 20 other SFE fish species (Baerwald et al., 2011). Assay sensitivity and precision were assessed using the Limit of Detection (LOD) and Limit of Quantification (LOQ) following standardized methods for analysis of eDNA samples using qPCR (Klymus et al., 2020; Merkes et al., 2019). The LOD was defined as the lowest concentration in which the target molecule can be detected in 95% of replicates (Bustin et al., 2009; Klymus et al., 2020). The LOQ was defined as the lowest concentration at which the coefficient of variation (CV) of the threshold cycle (Ct, also known as quantification cycle, Cq) value is less than 35% (Kubista, 2014; Forootan et al., 2017; Klymus et al., 2020).

**Table 2:** eDNA detection of delta smelt.

Primer or probe	Sequence type	Sequence (5′–3′)	Reporter	Quencher
CytB-Htr-F	Forward primer	AATGGCCAACCTTCGGAAA	–	–
CytB-Htr-R	Reverse primer	GARATATTRGAGGGTGCAGG	–	–
CytB-Htr-P	Probe	CCCATCCCCTCCTGAAAATTACCAACG	6 FAM	BHQ

**Note:**

Primers and probe previously validated for detection of delta smelt DNA in predator guts (Baerwald et al., 2011) were used in this study.

A 12-point dilution series of a 157-bp synthetic oligonucleotide gBlocks Gene Fragment (Integrated DNA Technologies, San Diego, CA, USA) of the delta smelt cytochrome b gene amplified was used to calculate a standard curve. The four-fold dilution series started at ~3.5 × 10^6^ copies/reaction (~5.74 × 10^5^ copies/µL) and ended at 0.84 copies/reaction (0.14 copies/µl; File S2). qPCR was performed in a 20 µL total volume containing: 1 µL gBlocks template at the appropriate dilution, 0.9 µM final concentration of each primer, 0.9 µM final concentration of the probe, and 2× TaqMan Environmental Master Mix 2.0 (Applied Biosystems, Waltham, MA, USA). qPCR was conducted on a single CFX Touch Real-Time PCR instrument (Bio-Rad Laboratories, Hercules, CA, USA) with the delta smelt target reported on the FAM channel and amplification curves checked by eye. Thermocycling conditions are provided in Table 3.

**Table 3:** Thermocycling protocol for delta smelt eDNA detection.

Step	Time	Temperature	Cycles
Initial denaturation	10 min	95 °C	1
Denaturation	15 s	95 °C	50
Annealing/Extension	1 min	63 °C	

The LOD and LOQ were calculated in R version 4.1.3 using the Generic qPCR Limit of Detection (LOD)/Limit of Quantification (LOQ) calculator (Merkes et al., 2019). A two-parameter Weibull type two function was selected by the calculator as the best fitting model (lack of fit test: *P* = 0.999999999999999) and was used to calculate effective LODs (DNA copies/qPCR reaction) for two to eight qPCR replicates. The effective LOD decreases as the number of qPCR replicates increases (Klymus et al., 2020).

### Field sampling

**Figure 2:**
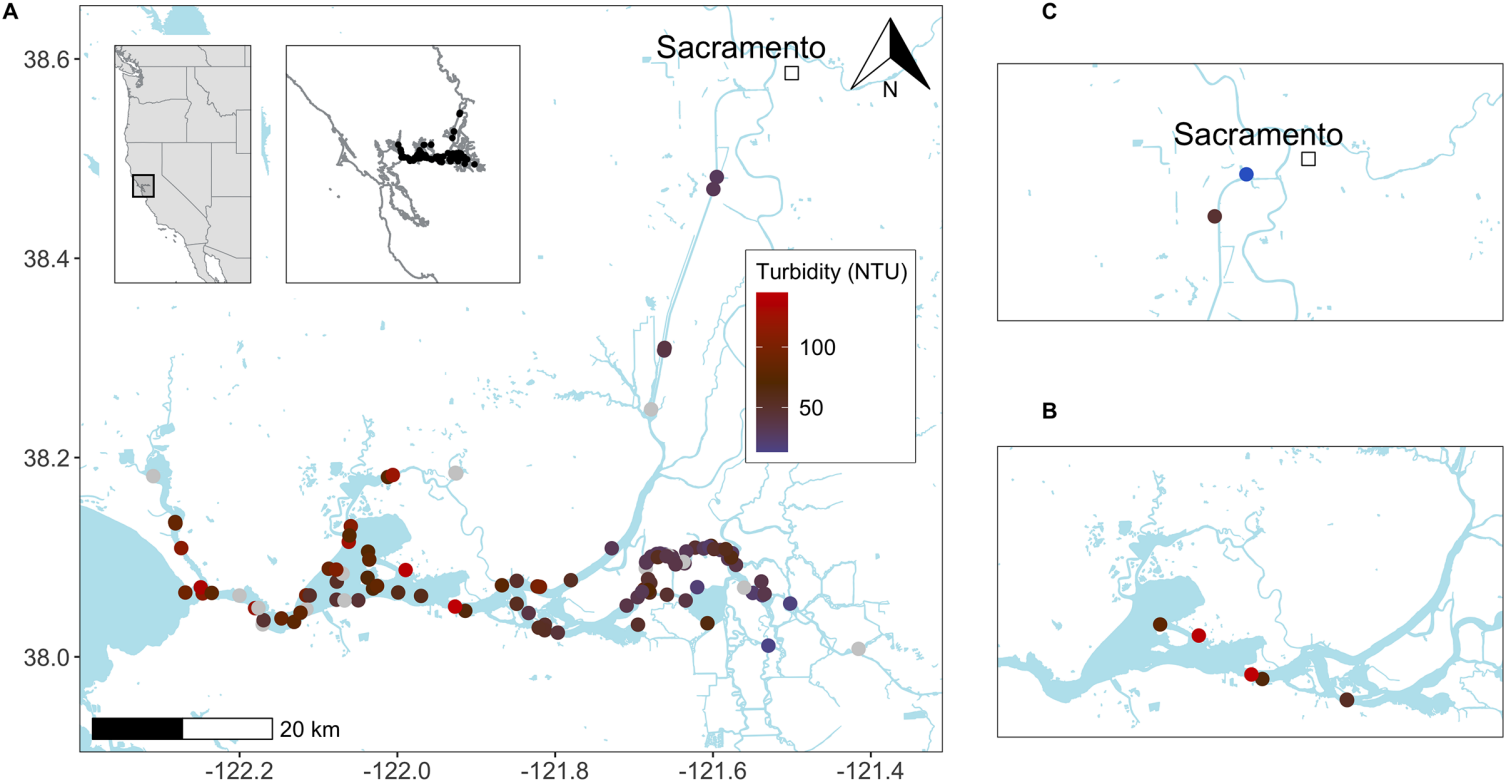
Delta smelt trawl and eDNA studies in the upper San Francisco Estuary (California, USA). Sites where delta smelt were detected by (A) Enhanced Delta Smelt Monitoring (EDSM; USFWS 2019) trawl surveys and (B) trawl surveys paired with eDNA sampling (for turbidity values, see File S7 and S4, respectively). Collection sites (C) for non-turbid water (~5 NTU) from the upper Sacramento Deep Water Shipping Channel (DWSC; 38.5653, −121.5539) and turbid water (~50 NTU) from Prospect Slough, just west of the DWSC (35.5299, −121.589) used in the filtration experiment (Fig. 3). Base maps from the Conservation Lands Network regional biodiversity GIS database (https://purl.stanford.edu/qh320kj0191).

Field samples (*n* = 32) for eDNA analysis were collected during Enhanced Delta Smelt Monitoring (EDSM) surveys targeting adult delta smelt in the San Francisco Estuary (SFE; CA, USA) in winter 2017 (File S3; Fig. 2). One-liter water samples were collected at 0–1 m depth using the peristaltic pump during or immediately following a 5-min trawl deployment. Dedicated personnel conducted eDNA sampling and gear was physically separated from trawling gear and activity. Field samples were placed in sterile zipper re-closable plastic bags and stored on ice in a dedicated cooler until filtration (<12 h). Field negative control samples of deionized water were transported to the field and processed with the field samples.

**Figure 3:**
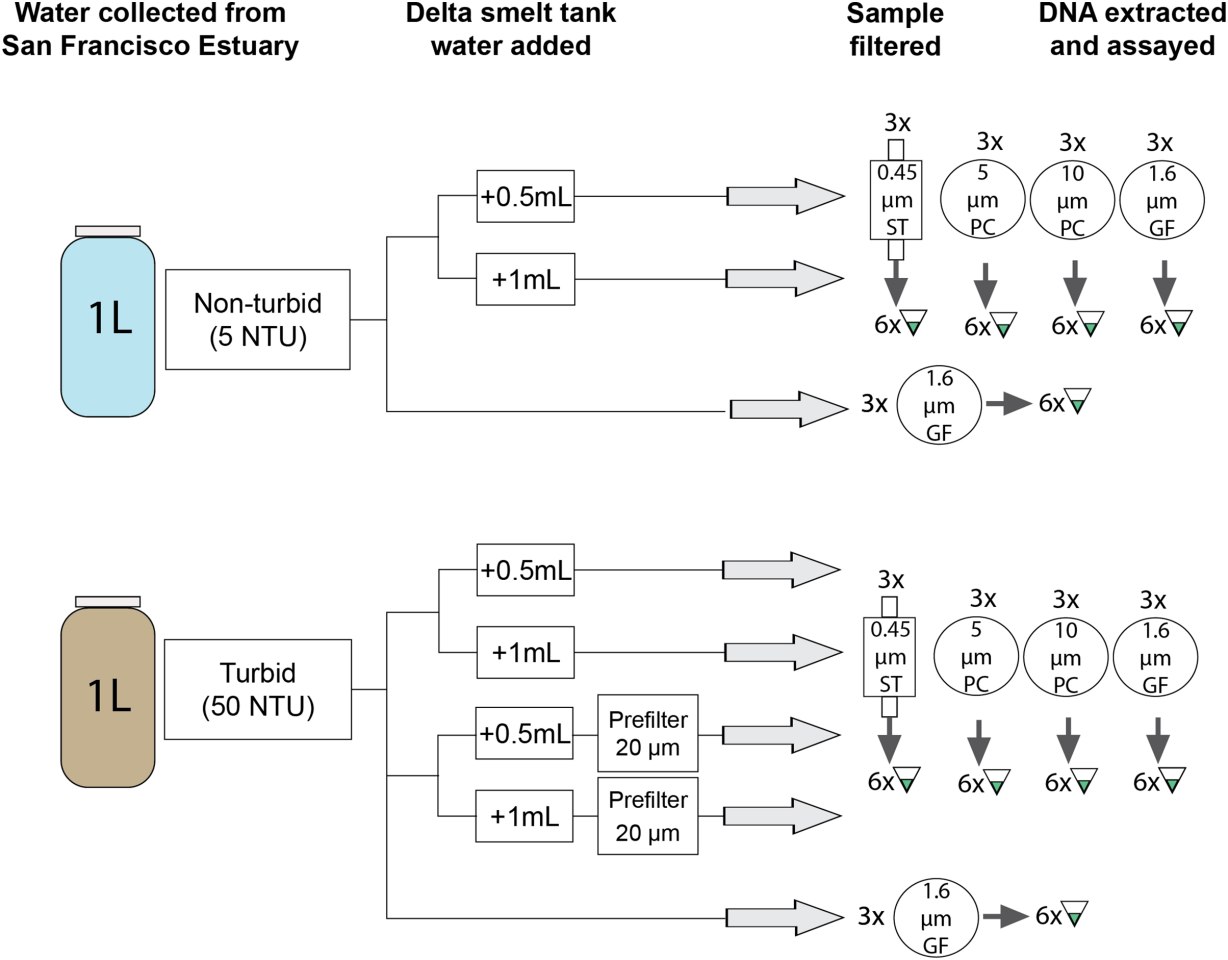
Schematic representation of experimental work. Three biological replicates of six treatments were each filtered on four different filter types and assessed in 6 qPCR replicates using a species-specific assay (Baerwald et al., 2011). GF, glass fiber filter; PC, polycarbonate filter; ST, Sterivex cartridge filter.

Water samples were filtered in the field or in a laboratory space free of delta smelt and delta smelt DNA. Samples were filtered using 1.6 μm glass fiber filters (Table 1) in sterile filter holders (Swinnex-47, MilliporeSigma, Burlington, MA, USA) attached to sterilized silicon tubing. Each 47 mm filter was folded twice lengthwise and placed in a sterile 2 mL tube. The tubes were placed in individual sterile plastic bags and immediately frozen on dry ice. Samples were stored at −20 °C for less than 4 weeks until extraction.

### Experimental work

Experiments used non-turbid and turbid water from the SFE and manually added eDNA from delta smelt tanks (Fig. 3). The turbidity value of the turbid water was selected to be similar to conditions encountered during the field survey (File S3). First, estuarine water was collected in sterilized 5-gal buckets from sites where delta smelt are not typically present (Fig. 2). Turbidity was measured at ~5 NTU (“non-turbid”) and ~50 NTU (“turbid”) with a 2100Q Portable Turbidimeter (Hach Company, Loveland, CO, USA). The buckets were covered and transported at ambient temperatures to the University of California-Davis (UC Davis) campus (Davis, CA, USA). Water was homogenized by stirring with a sterilized implement before transfer to sterilized 1-L bottles.

Tank water was collected from a delta smelt tank at the UC Davis Center for Aquatic Biology and Aquaculture in a sterile 1-L bottle and transported on ice to the laboratory. The 340-L tank (recirculating aquaculture system with daily make-up water to maintain a tank volume) contained approximately 186 captive-bred adult delta smelt sourced from the UC Davis Fish Conservation and Culture Laboratory (Byron, CA, USA) for research unrelated to this study. Tank water was used instead of DNA extract or a synthetic oligonucleotide because natural eDNA particle size and properties (*e.g*., stickiness) may influence detection probability (Turner et al., 2014; Thomas et al., 2018; Barnes et al., 2021). The tank water sample was homogenized using several gentle inversions and 0.5 mL was pipetted into each 1 L bottle of estuarine water to produce concentrations (as assessed by Ct value) similar to field samples (File S4). To be conservative, the same process was repeated using 1 mL added to each bottle in case 0.5 mL tank water was undetectable in some sample types. Adding two small but different volumes of tank water also provided an opportunity to assess if small differences in low concentration eDNA samples can be distinguished. Bottles were placed in a sterilized cooler with ice for up to 8 h before filtering.

Experimental samples were then filtered using an overall set-up similar to the field sampling. Four filter types were selected to capture fish mitochondrial DNA in the 1–10 µm size range where they are most abundant (Table 1; Turner et al., 2014; Wilcox et al., 2015). Glass fiber and polycarbonate filters were loaded into sterile filter holders attached to silicon tubing, as with the field samples. Cartridge filters were attached directly to the tubing. Nylon mesh filters (used as prefilters) were also loaded into sterile filter holders; estuarine water with tank water added was pumped through the prefilter prior to capture with the end filter. Three biological replicates were filtered using each of the four filter types and three treatments: non-turbid water, turbid water, and turbid water with the addition of a prefilter, resulting in 72 1-L biological replicates (Fig. 3). Prior to filtering, each bottle was gently inverted several times. Samples were pumped through filters until the entire 1-L sample was filtered or flow ceased (a maximum filtration time ~15 min); filtration volumes less than 1 L reflect clogging caused by particulate matter.

Each 47 mm filter was preserved and stored as described for field sampling. Cartridge filers were capped at both ends and preserved using the same protocol. Three negative control samples of 5 NTU and 10 NTU estuarine water without tank water were processed with the field samples. Samples were stored at −20 °C for less than 2 weeks until extraction.

### DNA isolation and qPCR analysis

DNA from whole 47 mm filters was extracted in a dedicated eDNA laboratory space. DNA was extracted from field samples following the manufacturer’s protocol for the DNeasy Blood and Tissue Kit (Qiagen, Hilden, Germany) and processed using the Zymo OneStep PCR Inhibitor Removal Kit (Zymo Research, Irvine, CA, USA). DNA from experimental samples was extracted using the DNeasy PowerWater Kit (Qiagen, Hilden, Germany), which has been shown to effectively remove PCR inhibitors (Eichmiller, Miller & Sorensen, 2016). DNA from cartridge filters was extracted using the DNeasy PowerWater Sterivex Kit (Qiagen, Hilden, Germany). The extraction protocol for both PowerWater kits followed the manufacturer’s instructions including the optional heat lysis. The final elution volume was 100 µl into LoBind tubes (Eppendorf, Hamburg, Germany) with an extended incubation time to increase DNA yield (File S5).

Each biological replicate (DNA extracted from a single filter) was assayed for delta smelt in multiple technical replicates (qPCR reactions); eight replicates were used for field samples and six replicates were used for experimental samples. qPCR methods followed the methods described in for assay validation except that a larger volume of DNA template (6.1 µL) was added to each reaction. No-template negative controls and gBlocks positive controls were included on each qPCR plate.

### PCR inhibition testing

Turbid water used in the experiment was tested for PCR inhibition using TaqMan Exogenous Internal Positive Control (IPC) Reagents (Applied Biosystems, Waltham, MA, USA), delta smelt primers and probe (Baerwald et al., 2011), and TaqMan Environmental Master Mix 2.0. qPCR was performed in a 20 µL volume with reagent volumes as previously described; IPC reagent volumes and cycling conditions followed manufacturer recommendations. Two template types were tested in triplicate reactions: 1-L negative control turbid estuarine water (50 NTU) and 1-L positive control of filtered delta smelt tank water. Both samples were filtered on glass fiber filters (1.6-µm pore) and extracted using the DNeasy PowerWater Kit as described above. Negative controls without both IPC and delta smelt target (*n* = 6) and with IPC target but without delta smelt target (*n* = 6) were included on the same plate. The IPC target was reported on the CFX Touch Real-Time PCR instrument’s HEX channel (equivalent to VIC) and the delta smelt target was reported on the FAM channel as above. Amplification of the IPC target was evaluated qualitatively for a shift in Ct value; Ct values that increase >3 cycles beyond no-template controls or do not amplify are considered inhibited (Hartman, Coyne & Norwood, 2005).

### Detection criteria and censored data methods

qPCR reactions generating a Ct value less than the LOD were considered positive detections. Due to low detection success in field samples, no further analysis was conducted. For the experimental samples, detection was evaluated as presence/absence and using eDNA copy numbers. Amplification of 50% of the technical replicates (*n* = 6) was the threshold for determining a positive biological replicate. Copies per liter of water filtered (copies/L) of each biological replicate was calculated using a censored data method.

Censored data methods enable robust statistical analysis of datasets with missing observations or observations that fall below a limit of detection (Helsel, 2012). Ct values for technical replicates that did not amplify or that generated a Ct value above the LOD were imputed using the R package nondetects, a censored data method (McCall et al., 2014). Imputed Ct values were used for further analysis of eDNA concentrations.

**Figure 4:**
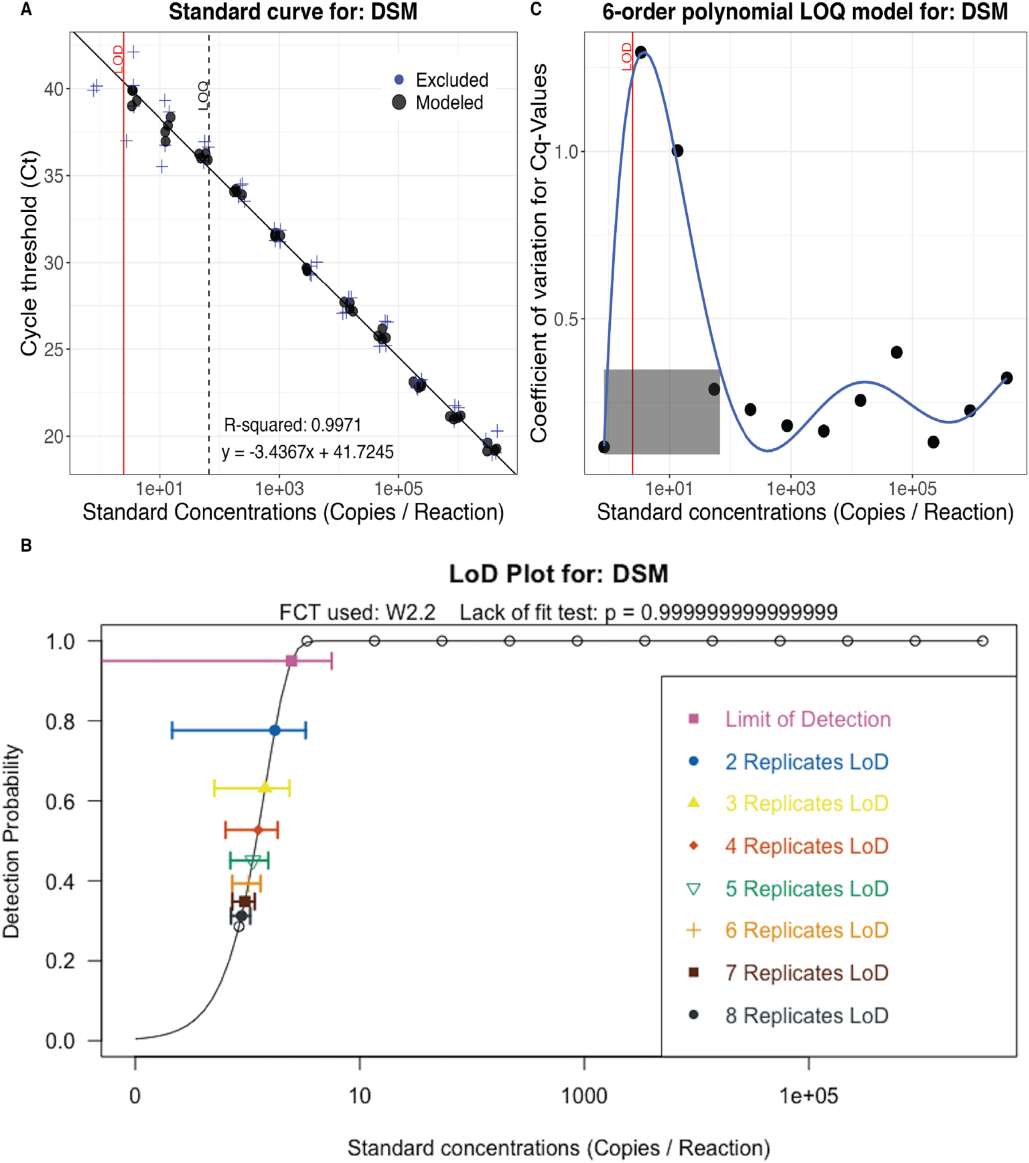
eDNA assay validation. Calibration curve (A), effective Limit of Detection (LOD) for up to eight technical replicates (B), and Limit of Quantification (LOQ) model (C) for the delta smelt assay (Baerwald et al., 2011). Calculations and data visualization follow standard methods for validating eDNA assays (Klymus et al., 2020; Merkes et al., 2019).

DNA copies/reaction were calculated from observed and imputed Ct values using the standard curve (Fig. 4) and adjusted for volume filtered using the following equation:

$\left( {{{{\mathrm{Target\, copies\, detected\, in\, qPCR\, reaction}}} \over {{\mathrm{DNA\, extraction\, volume\, used\, in\, qPCR\, reaction}}}}} \right) \times \left( {{{{\mathrm{DNA\, extraction\, total\, volume}}} \over {{\mathrm{Water\, volume\, filtered\, per\, sample}}}}} \right)$

$= \left( {{{\# {\mathrm{ copies}}/{\mathrm{reaction}}} \over {6.1{\rm \,\mu} {\mathrm{l/reaction}}}}} \right) \times \left( {{{100{\rm \,\mu} {\mathrm{l/sample}}} \over {0.1{\mathrm{ \,to\, }}1{\,\mathrm{L}}/{\mathrm{sample}}}}} \right) = {{{\mathrm{copies}}} \over {\mathrm{L}}}$

DNA copies/L for each biological replicate was calculated from the mean Ct of technical replicates. The LOD and LOQ were adjusted to copies/L for the actual water volume filtered in each sample.

### Statistical analysis

Statistical analysis evaluated qPCR results under the null hypothesis that turbidity conditions and filtration methods had no significant influence on target presence/absence or DNA copy number detected. Statistical tests were conducted in R (R Core Team, 2021) and significance was evaluated at alpha level 0.05. Fisher’s exact tests were used to compare the presence/absence in turbid samples (with and without a prefiltration step) with non-turbid samples. Comparisons between filter types were not made because all samples were positive in non-turbid water. For comparisons of eDNA copy number, a Kruskal-Wallis rank sum test was used to compare copies/L between filter types in non-turbid water. Following a significant Kruskal-Wallis test, Dunn’s test of multiple comparisons was applied to determine which groups were significantly different. Due to significant differences among filter types, comparisons between non-turbid, turbid and prefiltered turbid samples were made by filter type (*n* = 4).

Detection success was further evaluated using a model comparison approach implemented in the R packages lme4 (Bates et al., 2015) and bbmle (Booker & R Development Core Team, 2022). Models included five covariates as fixed effects: “turbidity” (a categorical variable with two levels corresponding to 5 NTU or 50 NTU); “filter type” (a categorical variable with four levels corresponding to the four filter types); “prefilter” (a categorical variable with two levels), “volume filtered” (a continuous variable of the volume of water filtered rounded to the nearest 50 mL), and “volume of tank water added” (a categorical variable with two levels corresponding to addition of 0.5 or 1 mL of water from the tank of delta smelt) and potential interactions.

Target presence/absence in each technical replicate was the response variable in generalized linear mixed models (GLMMs) fitted using a binomial distribution. Biological replicate was included as a random effect because technical replicates from the sample bottle are not independent observations. Copies/L calculated using a censored data method was the response variable in generalized linear models (GLMs) fitted using a negative binomial distribution because initial model fitting with a Poisson distribution indicated significant overdispersion of residuals. Models were compared using Akaike’s Information Criterion (AICc), a measure of goodness-of-fit that is corrected for small sample size, and the best model was selected. AICc values are useful for evaluating model predictive power because they account for the tradeoff between model fit and complexity (Harrison et al., 2018). R code is available at in the open access repository: https://github.com/annholmes/eDNA-experiments-in-turbid-water.

## Results

### Assay performance

PCR efficiency calculated using the serial dilution (Fig. 4; File S2) was 95%. The one replicate Limit of Detection (LOD) was 2.47 (±1.59) copies per PCR reaction and the Limit of Quantification (LOQ) was 67 copies per PCR reaction (Fig. 4). Although the calculated LOD value is below the theoretical minimum LOD of three copies, it is valid because three is included in the 95% confidence interval (Wittwer & Kusukawa, 2011; Forootan et al., 2017). The effective LOD for six technical replicates was 1.02 copy per PCR reaction (±0.15; Fig. 4).

### PCR inhibition testing

No PCR inhibition was detected in internal positive control (IPC) samples with turbid water (mean Ct 26.84) compared with no template controls (mean Ct 26.97) and delta smelt tank water samples (mean Ct 27.04). Complete results are reported in File S6.

### Delta smelt detection in the field

Delta smelt DNA was detected in one technical replicate at four sites where the Enhanced Delta Smelt Monitoring (EDSM) trawl also detected delta smelt (File S4). Mean turbidity for all eDNA field samples and field samples with positive detections was 90 NTU and 102 NTU, respectively (Files S3 and S4). Mean turbidity of 112 EDSM trawls that detected at least one delta smelt (median = 1 delta smelt, mean = 1.4 delta smelt) from December 2016 to March 2017 was 58 NTU (range: 13-145 NTU; Fig. 2; File S7; U. S. Fish and Wildlife Service et al., 2019).

### Delta smelt detection in experimental samples

**Figure 5:**
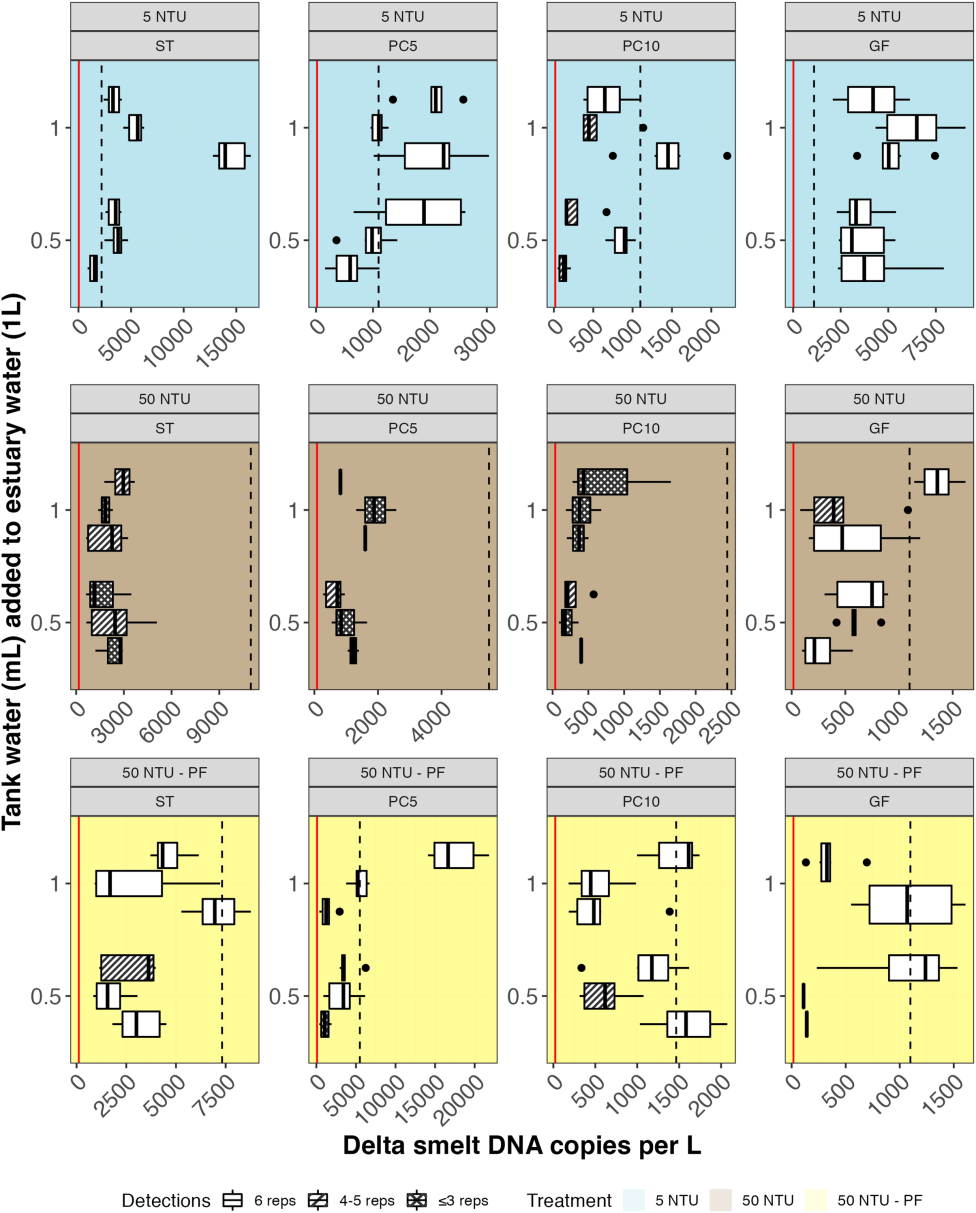
Delta smelt DNA copies/L detected in experimental samples by treatment (rows) and filter type (column). Six technical replicates were assayed for each of three biological replicates in three treatments: non-turbid estuary water (5 NTU; blue), turbid estuary water (50 NTU; brown), and turbid estuary water with prefiltration (50 NTU – PF; yellow). Either 0.5 or 1.0 mL of water from a delta smelt tank was added to each 1-L biological replicate. Unshaded boxes represent samples where 100% of technical replicates detected delta smelt. The volume-adjusted Limit of Detection (LOD) and Limit of Quantification (LOQ) are shown as red and dashed black lines, respectively. GF, glass fiber filter; PC, polycarbonate filter; ST, Sterivex cartridge filter.

Delta smelt DNA was detected in at least one of six technical replicates in 71 of 72 biological replicates (filters); 54 samples met the criteria for a positive sample with at least 50% of technical replicates detecting delta smelt (Fig. 5; Table 4; File S8). Seventy-six qPCR technical replicates (17.8%) did not amplify or generated a Ct value exceeding the LOD (Ct 40.38) and Ct values were imputed using the R package nondetects (McCall et al., 2014). Delta smelt DNA was not detected in negative control samples of estuarine water or in qPCR no template controls.

**Table 4:** Summary of eDNA detection results.

Treatment	Detection	DNA copies per reaction (6.1 µl template)					Approximate volume (L) filtered for each biological replicate	Mean DNA copies/L adjusted for volume filtered
*Non-turbid*	# Positive replicates (three biological replicates, six qPCR replicates each)	Mean	Median	SD	Min	Max		
GF+500	18/18	230.92	201.72	92.87	139.88	483.11	1	3,785.56
PC10+500	16/18	24.94	11.43	22.11	2.89	63.45	1	4,08.92
PC5+500	18/18	68.22	60.01	45.63	9.63	159.94	1	1,118.43
ST+500	18/18	86.45	92.12	36.12	26.73	143.68	0.50	2,834.28
GF+1000	18/18	320.78	306.50	110.07	127.36	552.38	1	5,258.72
PC10+1000	17/18	54.16	48.11	33.04	20.31	134.37	1	887.93
PC5+1000	18/18	105.65	102.78	39.91	58.55	185.34	1	1,732.01
ST+1000	18/18	235.37	171.71	154.77	73.03	499.57	0.50	7,717.17
*Turbid without prefilter*								
GF+500	18/18	30.77	34.6	15.72	5.92	54.75	1	504.41
PC10+500	8/18	4.53	2.62	3.93	1.87	15.75	0.45	165.07
PC5+500	10/18	7.09	3.95	5.5	2.65	20.18	0.20	580.74
ST+500	11/18	9.45	4.98	8.53	2.63	30.98	0.10	1,548.39
GF+1000	17/18	46.89	47.26	33.24	3.11	98.73	1	768.76
PC10+1000	9/18	8.20	3.72	10.55	2.01	45.39	0.45	298.88
PC5+1000	5/18	6.71	1.43	9.32	1.24	31.4	0.20	550.23
ST+1000	12/18	9.24	6.52	7.54	1.76	22.46	0.10	1,514.80
*Turbid with prefilter*								
GF+500	9/18	24.32	4.75	33.9	2.05	93.58	1	398.65
PC10+500	17/18	49.46	48.86	26.31	6.33	94.84	0.75	1,081.08
PC5+500	17/18	32.22	36.76	22.5	4.71	76.03	0.20	2,640.61
ST+500	17/18	22.22	20.45	11.93	5.84	41.32	0.15	2,428.84
GF+1000	9/18	29.22	19.91	32.76	0	98.07	1	478.96
PC10+1000	17/18	38.65	29.03	25.53	8.33	79.68	0.75	844.90
PC5+1000	17/18	98.52	63.87	88.61	4.65	266.12	0.20	8,075.07
ST+1000	17/18	44.53	46.64	22.07	8.39	80.21	0.15	4,867.00

**Note:**

Results of the filtration experiment using non-turbid water, turbid water, and turbid water plus a prefilter. Detailed results are in File S8. GF, glass fiber filter; PC, polycarbonate filter; ST, Sterivex PVDF filter.

In non-turbid water, nearly 100% of technical replicates detected delta smelt DNA (Fig. 5; Table 4). In turbid water, only glass fiber filters had 100% detection. In turbid water with the addition of a prefilter, 5um polycarbonate and cartridge filters generated all positive samples (>50% detection in technical replicates). Statistical comparisons of the number of positive samples did not show significant differences according to turbidity and prefiltration; comparisons were not made based on filter type because of all samples were positive in non-turbid water.

eDNA copies per liter of water filtered (copies/L) were highest for glass fiber filters and cartridge filters (Fig. 5; Table 4; File S8). Water volume filtered was lower for cartridge filters and for all turbid samples except those using glass fiber filters. Statistical comparisons of copies/L between filter type for non-turbid water samples identified two significant differences: glass fiber filters to 10 μm polycarbonate filters and cartridge filters to 10 μm polycarbonate filters. Within filter type, significant differences in copies/L between non-turbid and turbid water without a prefilter were identified in both glass fiber filters and cartridge filters. A significant difference in copies/L was identified between non-turbid and prefiltered water for glass fiber filters. Compared with turbid water sampled without prefilter, significantly more copies/L were detected for both types of polycarbonate filters (5 and 10 μm) when a prefilter was used.

The full model for delta smelt presence/absence identified turbidity as the only significant effect; the best model selected using AICc comparison included turbidity and prefilter (Table 5). The full model for DNA copies/L identified turbidity, filter type, and an interaction between filter type and prefilter as significant effects; the same effects were included in the best model selected by AICc comparison (Table 5).

**Table 5:** Summary of eDNA detection model results.

		Full model: copy number				Best model: copy number			
		Estimate	SE	Z-value	*P*-value	Estimate	SE	Z-value	*P*-value
Intercept		8.348	0.358	23.320	<0.001	8.277	0.239	34.560	<0.001
Filter type	PC5	−1.372	0.473	−2.899	0.004	−0.677	0.289	−2.344	0.019
	PC10	−2.143	0.474	−4.525	<0.001	−1.525	0.289	−5.269	<0.001
	ST	−0.169	0.473	−0.358	0.721	0.460	0.289	1.591	0.112
Prefilter		−0.467	0.422	−1.107	0.268				
Turbidity		−0.039	0.009	−4.179	0.001	−0.029	0.005	−6.273	<0.001
Tank water added		0.555	0.399	1.390	0.164				
Filter type: Prefilter	GF					−0.768	0.369	−2.081	0.037
	PC5	2.497	0.533	4.685	<0.001	2.412	0.368	6.547	<0.001
	PC10	1.850	0.534	3.467	0.001	1.543	0.369	4.183	<0.001
	ST	1.158	0.533	2.173	0.030	0.891	0.368	2.418	0.016
Filter type: Turbidity	PC5	0.024	0.012	2.002	0.045				
	PC10	0.022	0.012	1.838	0.066				
	ST	0.019	0.012	1.606	0.108				
Filter type: Tank water added	PC5	0.186	0.435	0.427	0.669				
	PC10	0.067	0.435	0.153	0.878				
	ST	0.249	0.435	0.573	0.567				
Prefilter: Tank water added		0.204	0.377	0.542	0.588				
Turbidity: Tank water added		−0.009	0.008	−1.067	0.286				
**Goodness of fit**									
AICc	1,202.7								1,186.4
Degrees of freedom	19								10
		**Full model: replicate presence/absence**				**Best model: replicate presence/absenc**			
		**Estimate**	**SE**	**Z-value**	***P*-value**	**Estimate**	**SE**	**Z-value**	***P*-value**
Intercept		5.867	1.686	3.480	0.001	5.432	1.290	4.212	<0.001
Filter type	PC5	−0.450	1.412	−0.318	0.750				
	PC10	−1.172	1.397	−0.839	0.402				
	ST	0.288	1.454	0.198	0.843				
Prefilter		1.878	1.110	1.692	0.091	−0.092	0.030	−3.014	0.003
Turbidity		−0.096	0.031	−3.074	0.002	1.910	1.137	1.679	0.093
Tank water added		0.201	0.317	0.632	0.527				
**Goodness of fit**									
AICc	302.0								295.4
Degrees of freedom	10								6

**Note:**

Results of the filtration experiment using non-turbid water, turbid water, and turbid water plus a pre-filter. Detailed results are in File S8. GF, glass fiber filter; PC, polycarbonate filter; ST, Sterivex PVDF filter.

## Discussion

Our findings suggest that field detection of delta smelt is limited by both turbidity and low eDNA concentration. While the field eDNA detections were concordant with the positive trawl samples, weak signals of species presence should be interpreted with caution (Goldberg et al., 2016). Although negative controls provided no evidence of false positive detection, low-level contamination may be a concern give the proximity of eDNA collection to trawl sampling. The field results thus served as motivation for experimental work to better understand the dynamics of delta smelt eDNA detection. The species-specific assay previously used for direct detection of delta smelt DNA in predator guts (Baerwald et al., 2011) was validated for eDNA applications. The calculated LOD value and absence of PCR inhibition indicated that the eDNA assay is well-optimized and is not likely a contributing factor to low detection success. Experiments using ecologically relevant turbidity and eDNA concentrations levels found that turbidity reduced detection in both presence/absence and eDNA copies/L. The addition of a prefilter improved DNA copies/L detected for cartridge and polycarbonate (screen-type) filters but not for glass fiber (nominal pore) filters. In non-turbid water, glass fiber filters and cartridge filters detected the highest number of eDNA copies/L, suggesting that glass fiber filters are the most effective method across the conditions tested. Glass fiber filters are also the most cost-effective filter tested.

### Turbidity

Our results agree with previous work implicating turbidity as one of the most important predicters of eDNA detection success (Wineland et al., 2019). While some study systems may be amenable to deferring eDNA sampling during periods of high turbidity, turbid conditions are a key feature of delta smelt habitat during spawning migration when monitoring is crucial. In the absence of PCR inhibition, reduced sample volume is a suspected culprit of reduced detection in turbid water. A positive relationship between sample volume and detection has been previously demonstrated in turbid (Williams, Huyvaert & Piaggio, 2017) and non-turbid (Wilcox et al., 2015; Sepulveda et al., 2019; Bedwell & Goldberg, 2020) conditions, but other results show no relationship (Mächler et al., 2016). We did not find a relationship between sample volume and delta smelt detection.

Interactions of eDNA particles, particulate matter, and filtration methods may be an important consideration for turbid conditions. Pressure on the filter increases when filters clog and may cause cells or mitochondria of the target organism to burst or clumps of cells to break apart (Thomas et al., 2018). It is hypothesized that this action reduces the size of eDNA particles; smaller particles move through the filter and are less likely to be captured, decreasing detection (Thomas et al., 2018). Filters with larger pores are commonly recommended to reduce clogging in turbid samples (*e.g*., Turner et al., 2014). Our data does not provide adequate comparison of pore size in filters of the same material to recommend a larger pore size but does indicate that filters with nominal pores may perform better than screen-type filters in turbid water. Nominal pores are irregular depth filters that retain only a percentage of particles larger than the stated pore size using multiple layers that trap particles inside the matrix.

The effect of turbidity on detection may be influenced by particle type and turbidity value. Turbidity in river-dominated estuaries like the SFE is caused by river inputs of suspended particulates and resuspended bottom sediments (Cloern, 1987); findings from this study may not be relevant for other types of particles (*e.g*., phytoplankton). In experimental ponds, turbidity up to 60 NTU was positively associated with eDNA detection; eDNA is hypothesized to stick to phytoplankton, the primary cause of turbidity in the ponds (Barnes et al., 2021). In a study using sedimentation boxes in rivers, eDNA was hypothesized to settle with or bind to suspended particulate matter (Díaz et al., 2020). Cellular studies suggest that cells may be more likely to adhere to each other than to foreign material (Coman, 1961). Turbidity may be positively associated with eDNA detection if relatively higher turbidity provides more suitable habitat for the target species (Kumar et al., 2022). Turbidity values considered “high” may be habitat-specific (File S11). Few other eDNA studies have analyzed eDNA detection success in turbidity conditions near 50 NTU (but see Barnes et al., 2021 and Furlan et al., 2019).

### Interpretation of low signal eDNA results

Aside from turbidity, extremely low abundance and small body size likely contribute to low eDNA detection success of wild delta smelt. In trawl samples that detected delta smelt from December 2016 to March 2017, 73% of detections were a single individual (File S7). Delta smelt trawl detection rates continued to decline after the study period (U. S. Fish and Wildlife Service et al., 2019). In addition, the amount of eDNA shed into the surrounding environment is positively related to fish biomass (Klymus et al., 2015), and adult delt smelt are only 5–7 cm in length. Water mixing likely further dilutes eDNA in the environment (Laporte et al., 2020); vertical mixing in the SFE is complex process influenced by both freshwater input and tidal action (Cloern, 1987).

Low signal eDNA data presents a challenge for both statistical analysis and data interpretation. Although qPCR is sensitive enough to detect a single target DNA molecule (Kubista et al., 2006), imperfect detection within technical replicates is expected for low concentration eDNA samples (Klymus et al., 2020). Imperfect detection creates challenges for data analysis. Common practices for analysis of qPCR with imperfect detection includes eliminating nondetects or substituting other values (*e.g*., zero or the LOD); these approaches introduce bias in statistical analysis and increase the false negative rate (McCall et al., 2014; Safford et al., 2022). Censored data methods address the nondetects and facilitate robust statistical analysis (Helsel, 2012).

There is no standard censored data method for eDNA data analysis. qPCR data does not conform to the expectations of other types of environmental data (Burns & Valdivia, 2008; Kralik & Ricchi, 2017) for which censored data methods were developed. Notably, qPCR does not have a Limit of Blank (LOB; Klymus et al., 2020), which is necessary in conventional censored data methods. The LOB represents the background signal on a measurement, but blank samples do not have a signal in qPCR (Kralik & Ricchi, 2017). Contamination or artifacts can produce a false positive detection, but this is not a background signal. We impute values using the R package nondetects which was developed for gene expression analysis and is applicable to other types of qPCR data. Imputation is a censored data method that treats technical replicates that do not amplify as missing data and models the missing qPCR values (McCall et al., 2014). A single imputation step for missing Ct values allows for downstream analysis to proceed using DNA copies, a more intuitive data format compared with logarithmic scale Ct values.

Finally, the DNA concentration (copies/L) in experimental samples was often below the calculated Limit of Quantification (LOQ). Although consistent methods for calculating and reporting assay limits greatly increase our ability to use and understand low signal data (Klymus et al., 2020), there is currently no consensus for how to address these “borderline” values in eDNA analysis. Unlike LOD, LOQ is not covered in the Minimum Information for Publication of Quantitative Real-Time PCR Experiments (MIQE) guidelines (Bustin et al., 2009) and may defined as a measurement precision appropriate to the analytical goals of the study (Armbruster & Pry, 2008). We quantified copies/L for experimental samples, including values below the LOQ. The approach goes against previous recommendations that Ct values below the LOQ should not be quantified due to unacceptable level of imprecision (Klymus et al., 2020). The logarithmic relationship between Ct value and DNA copy number means that a high coefficient of variation (CV) in Ct values is not very large in terms of copies, and we find that these values are useful for understanding the experimental outcome. However, DNA concentration is probably not useful for estimating delta smelt biomass or relative abundance in natural settings.

### Future directions

Experimental work encompassing a wider range of conditions and consistent methods for measuring turbidity could help further our understanding of eDNA detection in challenging conditions. Characteristics of suspended particulates that influence turbidity measurements (Davies-Colley & Smith, 2001) may be an important consideration for eDNA detection. Corrections could be developed for reduced detection sensitivity in turbid conditions but may be specific to particle type or habitat. Consistent metrics for quantifying turbidity will help advance eDNA research in turbid environments. Optical turbidity, water clarity (*e.g*., Secchi depth), and suspended sediments are related but different properties of water (Davies-Colley & Smith, 2001; File S11). Optical turbidity is objective, repeatable, and accurate across a broad range of turbidity values and can be used in most water bodies (Pickering, 1976).

Centrifugation may be an alternative to filtration for eDNA sampling in turbid conditions (*e.g*., U. S. Fish and Wildlife Service, 2022). The advantages of centrifugation include collection of equal water volumes regardless of turbidity, decreased sample processing time, and decreased risk of sample cross-contamination. The primary disadvantage of centrifugation is a substantial reduction in water volume compared with filtration; reduced species detection due to small water volumes can be partially offset by increasing the number of replicates per site. In addition, sediment load can decrease detection sensitivity in both filtered and centrifuged samples (U. S. Fish and Wildlife Service, 2022).

Finally, eDNA surveys of rare species should also consider whether detection criteria using Ct value or number of positive replicates are too stringent. Detections in single positive technical replicates are treated with caution, as they do not provide strong evidence of species presence (Goldberg et al., 2016). This approach intended to reduce the risk of false positives may eliminate true positive detections of a rare target. Appropriate field and laboratory negative controls can increase confidence in rare detections, but the proportion of false positives to true positives will be higher; tolerance for false positive detections must increase when the target species is rare (Darling, Jerde & Sepulveda, 2021).

## Conclusion

Although eDNA detection sensitivity decreases in turbid conditions, our findings provide optimism that reliable and repeatable eDNA detection of rare species is possible when appropriate methods are used.

## Supplemental Information

10.7717/peerj.16453/supp-1 Supplemental Information 1 Enhanced Delta Smelt Monitoring trawl survey.

10.7717/peerj.16453/supp-2 Supplemental Information 2 Delta smelt serial dilution data.

10.7717/peerj.16453/supp-3 Supplemental Information 3 Delta smelt eDNA field sampling data.

10.7717/peerj.16453/supp-4 Supplemental Information 4 eDNA field detection results.

10.7717/peerj.16453/supp-5 Supplemental Information 5 Detailed filter extraction protocol.

10.7717/peerj.16453/supp-6 Supplemental Information 6 Results of turbid water PCR inhibition test.

10.7717/peerj.16453/supp-7 Supplemental Information 7 Summary of the delta smelt trawl survey. Enhanced Delta Smelt Monitoring (EDSM) trawl survey data from December 2016 to March 2017 (USFWS 2019). All sites where delta smelt were detected by trawl are included, irrespective of eDNA sample collection.

10.7717/peerj.16453/supp-8 Supplemental Information 8 Delta smelt eDNA qPCR results. Ct values for nondetects have been imputed using the R package ‘nondetects’ (McCall et al., 2014).

10.7717/peerj.16453/supp-9 Supplemental Information 9 Boxplots of residual values before and after imputation.

10.7717/peerj.16453/supp-10 Supplemental Information 10 Statistical comparisons of filtration experiment results.

10.7717/peerj.16453/supp-11 Supplemental Information 11 The relationship between optical turbidity and water clarity.

## Additional Information and Declarations

### Data Availability

The following information was supplied regarding data availability: The R script is available at GitHub: https://github.com/annholmes/eDNA-experiments-in-turbid-water.
