# Supplementary material for: Evaluating environmental DNA detection of a rare fish in turbid water using field and experimental approaches
Source: PeerJ. 2024 Jan 2;12:e16453. doi: 10.7717/peerj.16453 (PMC10768661; doi:10.7717/peerj.16453)
Supplement: Supplemental Information 1 [file peerj-12-16453-s001.docx]

**Supplemental File S1: Enhanced Delta Smelt Monitoring trawl survey**

The Enhanced Delta Smelt Monitoring (EDSM) program is the most comprehensive survey of delta smelt (*Hypomesus transpacificus*) across the entire range of the species in the San Francisco Estuary. The EDSM samples delta smelt year-round using changing gear types that target larval, juvenile, and adult life stages. eDNA samples were collected alongside the EDSM during the winter (December through March) when adult fish are sampled by Kodiak trawl as

they migrate to the upper estuary. The Kodiak trawl is a net towed between two small boats. Fish

are funneled to the cod end of the net and collect in a live box designed to minimize mortality and stress. At the conclusion of the timed tow, the live box is brough onto one of the boats. Any

fish collected in the live box are transferred to a tub of water. Fish ≥ 25 mm fork length (FL) are measured and identified to species. EDSM sampling methods are further detailed in U.S. Fish and Wildlife Service (2019) and Mahardja et al. (2021).

References

**Mahardja B, Mitchell L, Beakes M, Johnston C, Graham C, Goertler P, Barnard D, Castillo G, Matthias B. 2021.** Leveraging Delta Smelt Monitoring for Detecting Juvenile Chinook Salmon in the San Francisco Estuary. *San Francisco Estuary and Watershed Science*, 19(1), 1–24. <https://doi.org/10.15447/sfews.2021v19iss1art2>

**U.S. Fish and Wildlife Service, Johnston C, Lee S, Mahardja B, Speegle J, Barnard D. 2019.** USFWS: San Francisco Estuary Enhanced Delta Smelt Monitoring Program data, 2016–2019. Version 1. Environmental Data Initiative. [https://doi.org/10.6073/pasta/98bce400502fae3a6 b77b3e96f6d51e7](https://doi.org/10.6073/pasta/98bce400502fae3a6%20b77b3e96f6d51e7)
