# Supplementary material for: Evaluating environmental DNA detection of a rare fish in turbid water using field and experimental approaches
Source: PeerJ. 2024 Jan 2;12:e16453. doi: 10.7717/peerj.16453 (PMC10768661; doi:10.7717/peerj.16453)
Supplement: Supplemental Information 5 [file peerj-12-16453-s005.docx]

**Supplemental File 4: Detailed extraction protocol for glass fiber filters**

Qiagen Experienced User Protocol in regular text

***Modifications and notes in bold italics***

Important points before starting

- Solution PW1 must be warmed to 55°C for 5–10 min to dissolve precipitates prior to use. Solution PW1 should be used while still warm.
- If Solution PW3 has precipitate, heat to 55°C for 5–10 min to dissolve precipitate.
- Shake to mix Solution PW4 before use.
- Perform all centrifugation steps at room temperature (15–25°C).
- ***Preheat incubator to 65ºC.***

**Procedure**

***Steps 1-3 pertain to filtration and filter preservation; see main text for the methods used in this study. This protocol works best for extracting DNA from 15-20 filters at a time. Up to 30 filters can be extracted at a t***

Step 4. ***Remove tubes from freezer and allow filers to thaw.*** ***Using sterile forceps,*** insert each filter into a 5 ml PowerWater Bead Pro Tube. ***Using 1-2 sterile forceps, unfold filter inside the 5 ml tube and expose the side with captured material. This can be a tricky procedure and proper folding of filters after folding greatly improves this process.***

Step 5. Add 1 ml of ***warm*** Solution PW1 to ***filter surface inside*** the PowerWater Bead Pro Tube.

Note: For samples containing organisms that are difficult to lyse (e.g., fungi and algae) an additional heating step can be included. See Alternative Lysis Methods in the Troubleshooting Guide. ***Incubate for 10 min at 65ºC.***

Step 6. ***Tighten caps on 5 ml tubes after heat incubation.*** Secure the tube horizontally to a Vortex Adapter (cat. no. 13000-V1-5 or 13000-V1-15).

Step 7. Vortex at maximum speed for 5 min. ***(Centrifuge was not used in this protocol because the equipment was not available.)***

***Notes: During this step the glass fiber filter should fully break apart. Sometimes the supernatant gets foamy. Remove tubes from***

Step 8. Transfer the supernatant to a clean 2 ml collection tube (provided). Draw up the supernatant using a 1 ml pipette tip by placing it down into the beads. Note: Placing the pipette tip down into the beads is required. Pipette until you have removed all the supernatant.

***Notes: Qiagen protocol says expect to recover 600–650 µl of supernatant. As glass fiber filters hold liquid, the amount of supernatant recovered is generally 600-1200 µl. For turbid samples, the supernatant should reflect the color of the filter (usually greenish or brownish).***

Step 9. Centrifuge at 13,000 x g (~8000 rpm) for 1 min.

Step 10. Avoiding the pellet, transfer the supernatant to a clean 2 ml collection tube (provided).

Step 11. Add 200 µl of Solution IRS and vortex briefly to mix. Incubate at 2–8°C for 5 min. ***(Longer incubation times appear to have no effect on extraction efficiency; this incubation step is a good time to break, if needed.)***

Step 12. Centrifuge the tubes at 13,000 x g (~8000 rpm) for 1 min.

Step 13. Avoiding the pellet, transfer the supernatant to a clean 2 ml collection tube (provided).

Step 14. Add 650 µl of Solution PW3 and vortex briefly to mix.

Step 15. Load 650 µl of supernatant onto an MB Spin Column. Centrifuge at 13,000 x g (~8000 rpm) for 1 min. Discard the flow-through. Repeat until all the supernatant has been processed.

Step 16. Place the MB Spin Column Filter into a clean 2 ml collection tube (provided). ***Use the provided 1.5 ml collection tubes but manually remove caps to prevent them from coming off in the centrifuge.***

Step 17. Add 650 µl of Solution PW4 (shake before use). Centrifuge at 13,000 x g (~8000 rpm) for 1 min.

Step 18. Discard the flow-*through (or place into clean 2 ml collection tube)* and add 650 µl of ethanol (provided) and centrifuge at 13,000 x g (~8000 rpm) for 1 min. ***The bottom of the spin column is still in the liquid if using the collection tubes provided; discard the flow-through and centrifuge again at 13,000 x g (~8000 rpm) for 1 min.***

Step 19. Discard the flow-through and centrifuge again at 13,000 x g (~8000 rpm) for 2 min.

Step 20. Place the MB Spin Column into a clean 2 ml collection tube (provided).

***Note: It is important to use Eppendorf 1.5 ml LoBind tubes instead of provided collection tubes.***

Step 21. Add 100 µl of Solution EB to the center of the white filter membrane. ***Solution should be added carefully so that it sits on the membrane, not the side of the tube. Incubate for at least 20 min to increase DNA yield.***

Step 22. Centrifuge at 13,000 x g (~8000 rpm) for 1 min.

Step 23. Discard the MB Spin Column. The DNA is now ready for downstream applications. Note: We recommend storing DNA frozen (–90°C to –15°C) as Solution EB does not contain EDTA. To concentrate DNA, see the Troubleshooting Guide.
