# Supplementary material for: Evaluating environmental DNA detection of a rare fish in turbid water using field and experimental approaches
Source: PeerJ. 2024 Jan 2;12:e16453. doi: 10.7717/peerj.16453 (PMC10768661; doi:10.7717/peerj.16453)
Supplement: Supplemental Information 9 [file peerj-12-16453-s009.docx]

**Supplemental File S9: Comparison residuals of Ct values before and after imputation**


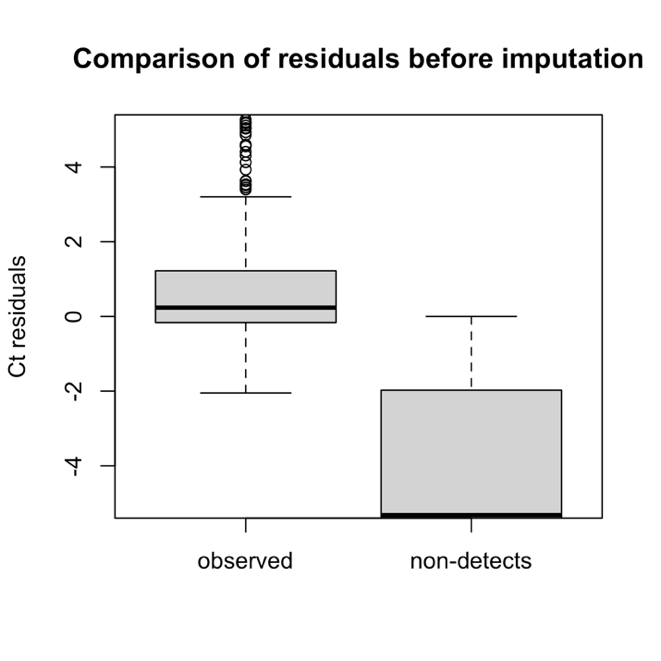

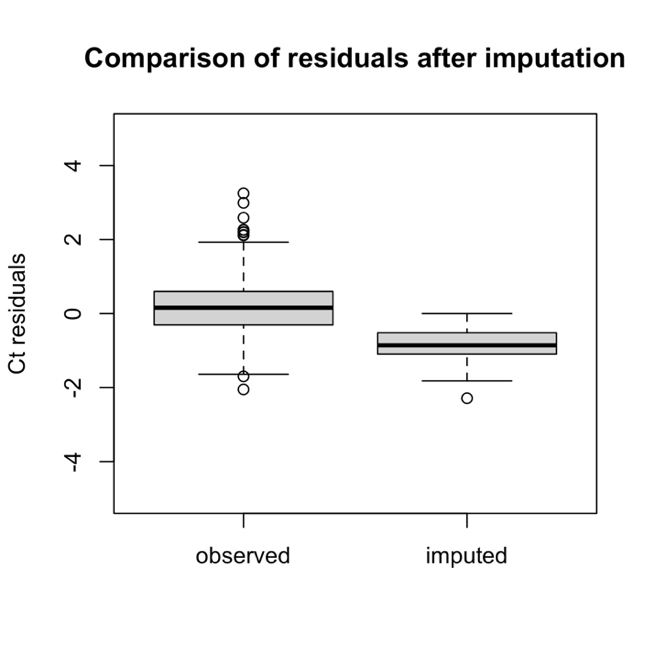


Following methods of McCall et al. (2014), replicate residuals of cycle threshold (Ct) values were calculated for observed values and non-detects before imputation (left) and for observed and imputed values after imputation (right). Before imputation, non-detect Ct values were set to the maximum number of cycles in the thermocycling protocol (50).

Reference

**McCall MN, McMurray HR, Land H, Almudevar A. 2014.** On non-detects in qPCR data. *Bioinformatics* 30(16):2310–2316 DOI 10.1093/bioinformatics/btu239.
