# Supplementary material for: Evaluating environmental DNA detection of a rare fish in turbid water using field and experimental approaches
Source: PeerJ. 2024 Jan 2;12:e16453. doi: 10.7717/peerj.16453 (PMC10768661; doi:10.7717/peerj.16453)
Supplement: Supplemental Information 10 [file peerj-12-16453-s010.docx]

**Supplementary File S10: Statistical tests**

**Presence/absence by sample:**

- **50% amplification of qPCR technical replicates = “positive sample”**

Number of samples considered positive for each sample type:

|  | GF | PC10 | PC5 | ST |
| --- | --- | --- | --- | --- |
| 5 NTU | 6 | 6 | 6 | 6 |
| 50 NTU | 6 | 5 | 3 | 6 |
| 50 NTU + PF | 3 | 6 | 4 | 6 |

No comparisons were made if all 6 samples met the criteria for a positive sample.

**Fisher’s exact test**

Contingency tables for Fisher’s test comparing presence/absence:

|  | + | - |
| --- | --- | --- |
| Non-turbid | 6 | 0 |
| Comparison | 5 | 1 |

**Not significant (p-value = 1)**

|  | + | - |
| --- | --- | --- |
| Non-turbid | 6 | 0 |
| Comparison | 4 | 2 |

**Not significant (p-value = 0.4545)**

|  | + | - |
| --- | --- | --- |
| Non-turbid | 6 | 0 |
| Comparison | 3 | 3 |

**Not significant (p-value = 0.1818)**

**Result: no significant differences (α = 0.05) in presence/absence across all comparisons of treatment and filter type.**

Note that if the requirement for samples to be considered “positive” is increased from 3 to 4 replicates, some of these comparisons become significant:

|  | GF | PC10 | PC5 | ST |
| --- | --- | --- | --- | --- |
| 5 NTU | 6 | 6 | 6 | 6 |
| 50 NTU | 6 | 1* | 1* | 3 |
| 50 NTU + PF | 3 | 6 | 4 | 6 |

*significant (α = 0.05)

Contingency tables for Fisher’s test:

|  | + | - |
| --- | --- | --- |
| Non-turbid | 6 | 0 |
| Turbid (w or w/o PF) | 1 | 5 |

**p-value = 0.01515**

|  | + | - |
| --- | --- | --- |
| Non-turbid | 6 | 0 |
| Turbid (w or w/o PF) | 4 | 2 |

**p-value = 0.4545**

**Delta smelt DNA copy number detected by sample:**

- Mean eDNA copies per L of water filtered of 6 technical replicates
- Non-detect values were imputed following McCall et al. (2014)

Test for normality to determine appropriate statistical test:

Shapiro-Wilk normality test:

W = 0.63103, **p-value = 3.648e-12**

(reject null hypothesis that the data is normal)

**Kruskal-Wallis test:**

Kruskal-Wallis chi-squared = 17.047, df = 3, **p-value = 0.0006913**

(reject hypothesis that the 4 groups are not different, but this test doesn’t tell us which groups are different)

**Dunn’s test:**

- Post-hoc test to determine which groups are different:
- multiple p-values show which groups are different; adjusted for multiple comparisons (Bonferroni correction)

| **Comparison** | **Z** | **P.unadj** | **P.adj** |
| --- | --- | --- | --- |
| GF - PC10 | 3.6334098 | 0.0002797003 | **0.001678202** |
| GF - PC5 | 2.4903146 | 0.0127630075 | 0.076578045 |
| PC10 - PC5 | -1.1430952 | 0.2529990615 | 1.000000000 |
| GF - ST | 0.5715476 | 0.5676284993 | 1.000000000 |
| PC10 - ST | -3.0618622 | 0.0021996471 | **0.013197882** |
| PC5 - ST | -1.9187670 | 0.0550138324 | 0.330082994 |

Some significant differences in eDNA copy number detected by filter type, so proceeded with separate statistical tests comparing treatment (non-turbid, turbid, and turbid + prefilter) within each filter type.

Glass fiber filters (GF):

Kruskal-Wallis chi-squared = 11.942, df = 2, **p-value = 0.002552**

Dunn’s test result:

| **Comparison** | **Z** | **P.unadj** | **P.adj** |
| --- | --- | --- | --- |
| 5 NTU - 50 NTU | 2.5414689 | 0.0110387756 | **0.033116327** |
| 5 NTU - 50 NTU - PF | 3.2985022 | 0.0009720211 | **0.002916063** |
| 50 NTU - 50 NTU - PF | 0.7570333 | 0.4490299133 | 1.000000000 |

Sterivex filters (ST):

Kruskal-Wallis chi-squared = 8.6667, df = 2, **p-value = 0.01312**

Dunn’s test result:

| **Comparison** | **Z** | **P.unadj** | **P.adj** |
| --- | --- | --- | --- |
| 5 NTU - 50 NTU | 2.7577642 | 0.005819817 | **0.01745945** |
| 5 NTU - 50 NTU - PF | 0.4866643 | 0.626496274 | 1.00000000 |
| 50 NTU - 50 NTU - PF | -2.2710999 | 0.023140931 | 0.06942279 |

Polycarbonate 5µm (PC5):

Kruskal-Wallis chi-squared = 10.398, df = 2, p-value = **0.005523**

Dunn’s test result:

| **Comparison** | **Z** | **P.unadj** | **P.adj** |
| --- | --- | --- | --- |
| 5 NTU - 50 NTU | 2.000731 | 0.045421402 | 0.136264205 |
| 5 NTU - 50 NTU - PF | -1.189624 | 0.234194305 | 0.702582915 |
| 50 NTU - 50 NTU - PF | -3.190355 | 0.001420983 | **0.004262949** |

Polycarbonate 10µm (PC10):

Kruskal-Wallis chi-squared = 8.6667, df = 2, p-value = **0.01312**

Dunn’s test result:

| **Comparison** | **Z** | **P.unadj** | **P.adj** |
| --- | --- | --- | --- |
| 5 NTU - 50 NTU | 1.784436 | 0.074352905 | 0.22305872 |
| 5 NTU - 50 NTU - PF | -1.135550 | 0.256144967 | 0.76843490 |
| 50 NTU - 50 NTU - PF | -2.919986 | 0.003500476 | **0.01050143** |

**Tests for overdispersion in linear models for copy number:**

**Using Poisson distribution:** DHARMa nonparametric dispersion test via mean deviance residual fitted vs.simulated-refitted **(**data: sim_poisson)

dispersion = 1381, p-value < 2.2e-16

alternative hypothesis: two.sided (suggests overdispersion is present)


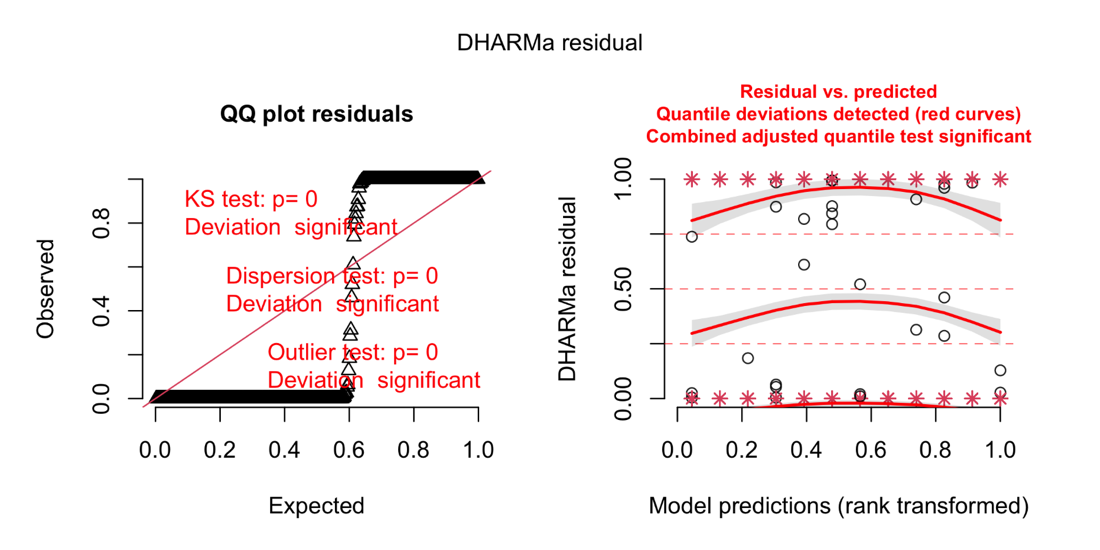


Results of the Poisson model indicate overdispersion (Left; dispersion = 1381, p-value < 2.2e-16). There are also major quantile deviations (Right).

**Using negative binomial distribution:** DHARMa nonparametric dispersion test via mean deviance residual fitted vs. simulated-refitted **(**data: sim_nb)

dispersion = 1.0019, p-value = 0.96

alternative hypothesis: two.sided (no overdispersion)


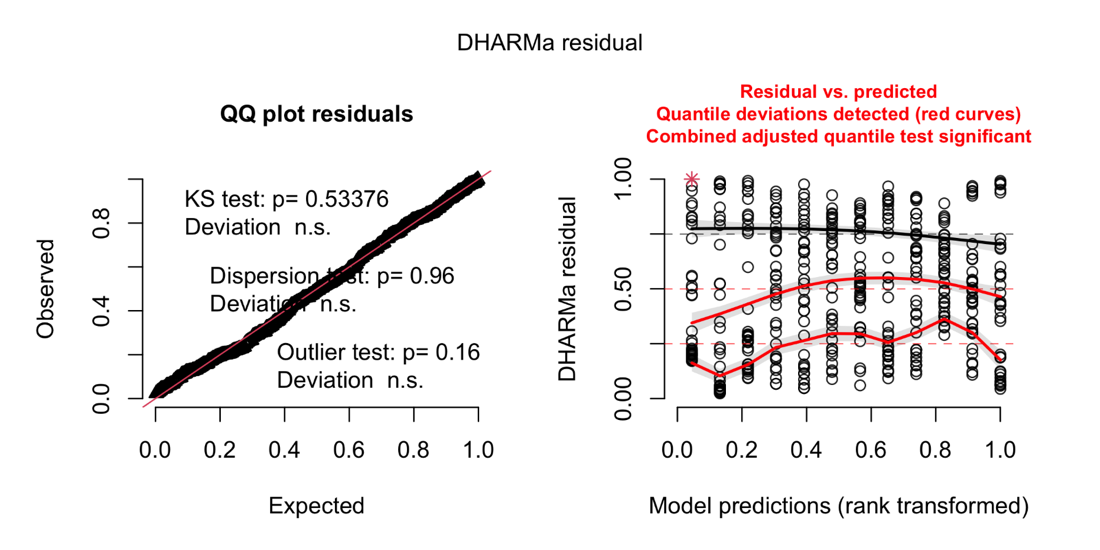


Results do not indicate overdispersion in the negative binomial model (Left; dispersion = 1.0019, p-value = 0.96). Some quantile deviations are detected in the residual vs. predicted plot (Right), but the plot is greatly improved from the previous model and generally acceptable.
