# Supplementary material for: Evaluating environmental DNA detection of a rare fish in turbid water using field and experimental approaches
Source: PeerJ. 2024 Jan 2;12:e16453. doi: 10.7717/peerj.16453 (PMC10768661; doi:10.7717/peerj.16453)
Supplement: Supplemental Information 11 [file peerj-12-16453-s011.docx]

**Supplemental File S10: The relationship between optical turbidity and water clarity**

Turbidity is a relative measurement and the definition of “high turbidity” justifiably varies greatly across different habitats and ecosystems. The concurrent use of units with imprecise relationships used to measure turbidity and related properties of water makes it more difficult to assess the impact of turbidity on eDNA detection in real-world scenarios. Optical turbidity (nephelometry or optical scattering) and Secchi depth (water clarity or transparency) are both reported in the literature as "turbidity." Optical turbidity measurements can be taken over a wider range of turbid conditions and habitat types compared with Secchi depth. For example, Secchi depth cannot be taken in high flow conditions or when ambient light is limited. However, the optical properties of particulates can influence turbidity readings and water clarity may have more ecological relevance (Davies-Colley and Smith 2007). Secchi depth measurements are a simple and inexpensive way to measure water clarity. Secchi depth measurements have some disadvantages such as the need to take measurements in situ and potential influences of human error and sunlight (Carlson and Simpson 1996).


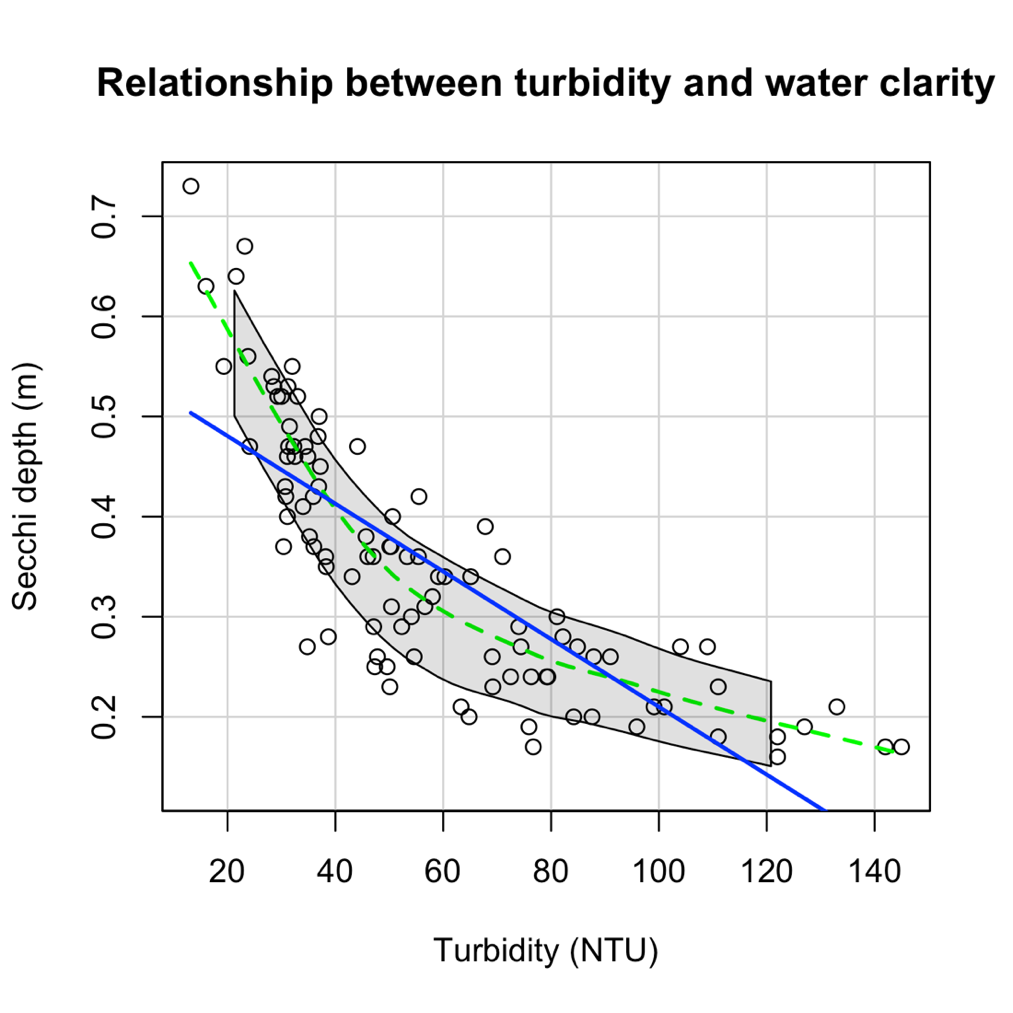


The relationship between real world optical turbidity values (NTU) and water clarity measured by Secchi depth (m) is plotted below; these data were recorded during winter Enhanced Delta Smelt Monitoring program surveys in the San Francisco Estuary (California, USA; Supplemental Files S1 and S7). Each dot (n=96) represents an individual tow where at least one delta smelt was detected, and both turbidity and Secchi depth were recorded. The relationship between turbidity and water clarity values is shown as a dashed green line; a regression line is shown in as a solid blue line. Using this general relationship, we can compare “high turbidity” from the SFE (this study) to “high turbidity” of inshore marine waters (Kumar et al. 2022) sampled for eDNA. The “high turbidity” Secchi depth measurement of 73 cm (Kumar et al. 2022) is approximately 10 NTU. The value is closer to this study’s “non-turbid water” (5 NTU) than to its “turbid water” (50 NTU).

References

**Carlson RE, Simpson, J. 1996.** A Coordinator's Guide to Volunteer Lake Monitoring Methods. North American Lake Management Society. 96 pp.

**Davies-Colley RJ, Smith DG. 2001.** Turbidity, suspended sediment, and water clarity: A review. *Journal of the American Water Resources Association*, 37, 1085-1101. <https://doi.org/10.1111/j.1752-1688.2001.tb03624.x>

**Kumar G, Farrell E, Reaume AM, Eble JA, Gaither MR. 2022.** One size does not fit all: Tuning eDNA protocols for high-and low-turbidity water sampling. *Environmental DNA* 4(1):167–180. <https://doi.org/10.1002/edn3.235>

**U.S. Fish and Wildlife Service, Johnston C, Lee S, Mahardja B, Speegle J, Barnard D. 2019.** USFWS: San Francisco Estuary Enhanced Delta Smelt Monitoring Program data, 2016–2019. Version 1. Environmental Data Initiative. [https://doi.org/10.6073/pasta/98bce400502fae3a6 b77b3e96f6d51e7](https://doi.org/10.6073/pasta/98bce400502fae3a6%20b77b3e96f6d51e7)
